# Characterizing the human hematopoietic CDome

**DOI:** 10.3389/fgene.2014.00331

**Published:** 2014-09-25

**Authors:** Mike Stein Barnkob, Christian Simon, Lars Rønn Olsen

**Affiliations:** ^1^Department of Clinical Immunology, Odense University Hospital, University of Southern DenmarkOdense, Denmark; ^2^Disease Systems Biology, Novo Nordisk Center for Protein Research, University of CopenhagenCopenhagen, Denmark; ^3^Department of Systems Biology, Center for Biological Sequence Analysis, Technical University of DenmarkLyngby, Denmark; ^4^Department of Biology, Bioinformatics Centre, University of CopenhagenCopenhagen, Denmark

**Keywords:** proteomics, immunotherapy, membrane proteins, CD molecules, flow cytometry, immunophenotyping

## Abstract

In this study, we performed extensive semi-automated data collection from the primary and secondary literature in an effort to characterize the expression of all membrane proteins within the CD scheme on hematopoietic cells. Utilizing over 6000 data points across 305 CD molecules on 206 cell types, we seek to give a preliminary characterization of the “human hematopoietic CDome.” We encountered severe gaps in the knowledge of CD protein expression, mostly resulting from incomplete and unstructured data generation, which we argue inhibit both basic research as well as therapies seeking to target membrane proteins. We detail these shortcomings and propose strategies to overcome these issues. Analyzing the available data, we explore the functional characteristics of the CD molecules both individually and across the groups of hematopoietic cells on which they are expressed. We compare protein and mRNA data for a subset of CD molecules, and explore cell functions in the context of CD protein expression. We find that the presence and function of CD molecules serve as good indicators for the overall function of the cells that express them, suggesting that increasing our knowledge about the cellular CDome may serve to stratify cells on a more functional level.

## Introduction

Proteins located in the cellular membrane are of particular interest in both research and therapy. Some membrane proteins are expressed throughout a cell's lifetime and are as such “cell defining,” while others are dynamically expressed and displayed on cell surfaces as a reflection of the current state or development of the cell. It has been estimated that approximately 20–26% of all genes encode surface proteins, many of which are currently under- or un-characterized (Cunha et al., 2009; Fagerberg et al., 2010).

The presence or absence of specific membrane proteins reflect both the internal state of the individual cell, as well as a cell's response to external stimuli (Cho and Stahelin, 2005). The extracellular domains of these proteins are heavily involved in cellular interactions, including cell-cell communication, and binding to the extracellular matrix as well as to peptides and hormones (Yamada, 1983; Plaut, 1987). For human blood cells especially, membrane proteins direct a number of important biological processes concerning development (Trowbridge and Thomas, 1994), decisions to proliferate (Ullman et al., 1990), cell motility, adhesion and homing (Carlos and Harlan, 1990; Luna and Hitt, 1992; Zarbock et al., 2011), and importantly, the activation, attenuation and inhibition of effector functions in immune cells (Bromley et al., 2001; Leibson, 2004). As such, defining and characterizing the dynamic expression of the plethora of membrane proteins across cell types in different states and diseases is fundamental for understanding their biological function, as well as finding disease biomarkers and drug targets.

Membrane proteins have also been used extensively to differentiate hematopoietic cells. Using flow cytometry, immunohistochemistry, mass spectrometry or mass cytometry to quantify the proportion of cells that express defining membrane proteins, it is possible to differentiate and thus quantify cell types within a cell population.

A common way to identify these proteins, sometimes referred to as surface markers, is by the cluster of differentiation (CD) scheme. Originally proposed in 1982 to help differentiate leukocytes and better classify monoclonal antibodies (Reinherz et al., 1986), it has since been used to characterize many other cell types (Zola et al., 2007). In the CD nomenclature, a surface marker that is recognized by several (a cluster of) antibodies is assigned a non-descriptive cluster of differentiation number. Since 1982, a number of workshops have continued to expand the list of validated CD molecules. Currently, 363 different proteins have been defined in the scheme, not including various subtypes of some CD molecules (Matesanz-Isabel et al., 2011).

While the CD repertoire has grown, efforts to deepen our knowledge of their expression across cell types and their interactions have not been able to follow pace. Furthermore, little is known about membrane proteins' concerted and dynamic co-expression patterns, making it difficult to take full advantage of many newer surface markers, in both basic research and therapies. For example, antibodies against CD52 were originally developed to remove T cells from donor bone marrow to prevent graft-vs.-host disease, but the marker was subsequently also found to be expressed on B cells (Hale et al., 1983), monocytes (Fabian et al., 1993) and dendritic cells (Buggins et al., 2002). More recently, off-target effects caused a deadly cytokine-storm in a patient treated with chimeric antigen receptor T cells aimed at CD340^+^ colon cancer cells, a target which was later found to be expressed on lung epithelial cells as well (Morgan et al., 2010). In another study, myelopoiesis was eradicated when researchers targeted CD123 on an acute myeloid leukemia (AML) cells (Gill et al., 2014). These cases exemplify the growing need for characterization of surface proteins, not only across cell types, but also throughout their developmental and disease states.

Traditionally, surface molecule expression is characterized at low rates using immunohistochemistry or flow cytometry, but in recent years new technologies such as mass spectrometry and mass cytometry has enabled the measurement of many parameters simultaneously. Recently mass-spectrometry have been used to characterize the whole proteome at the tissue level (Kim et al., 2014; Wilhelm et al., 2014), but at this point very little work has been done on individual human cell types. Membrane mass-spectrometry is disadvantaged by the fact that most surface proteins are hydrophobic and therefore poorly soluble, making analysis of this particular subset of proteins difficult (Sprenger and Jensen, 2010). This was highlighted in a recent proteomic study of the human tissue culture cell line U2OS, in which the authors found an under-representation of membrane proteins (Beck et al., 2011). Mass-cytometry is a novel hybrid mass-spectrometer and flow-cytometer, that utilizes metal isotopes as reporters. These can be coupled to known antibodies and because these isotypes can be detected without interference between channels, a large number of antibodies can be used—currently up to 44 per single cells (Bendall et al., 2012). The technique has been used to characterize 24 normal hematopoietic cell-types (Bendall et al., 2011), CD8^+^ cytotoxic T cells (Newell et al., 2012), NK cells (Horowitz et al., 2013) and most recently several B cell subsets (Bendall et al., 2014). At this point mass cytometry has not been used to characterize diseased cell types.

Another approach to characterizing the expression of surface proteins at high-throughput rates is to predict protein expression from gene expression data. In 2009, Gry et al. explored RNA and protein expression profiles for 1066 genes in 23 human cell lines, and found widely varying correlation. The mean correlation coefficient was 0.3, although for a subset of genes involved in the cytoskeleton, cellular maintenance and motility, a higher correlation was obtained. The reason for this discrepancy is most likely differences in transcription, translation, and mRNA and protein degradation rates. For example, it has been shown that proteins on average are five times as stable and 2800 times more abundant than their mRNA counterparts (Schwanhäusser et al., 2011), however little is known about the stability of membrane proteins in general.

Taken together, antibodies targeting proteins within the CD designation is still the most validated and most specific method for characterizing the membrane proteome in both normal and diseased hematopoietic cells. With current flow cytometry methods, normally only twelve markers can be assayed simultaneously (although it is technically feasible to measure up to 20 per cell), but vast amounts of these data have been generated historically. A few incomplete or now defunct CD molecules data resources have been reported (Díaz-Ramos et al., 2011), but no central resource for CD molecule data beyond basic gene information currently exists. Collecting, cleaning, organizing, and storing this data is undoubtedly a non-trivial task, but nonetheless one that may prove immensely valuable to both basic cell biology research by aiding functional classification of cells, cell stratification, but also to cancer immunotherapy and other therapeutics.

We here employ text mining techniques to extract a representative dataset, consisting of 6153 data points of CD protein expression across 206 hematopoietic cells, and utilizing these data, we analyze their concerted functionality across cell types.

## Materials and methods

### CD molecule protein and mRNA expression data for CDome analyses

All protein expression data used for analyses were extracted from experimental results described in the primary scientific literature accessed through PubMed. Additionally, data from secondary sources such as reviews, books, and conference proceedings were extracted.

All 279 known healthy and malignant human hematopoietic cells named in WHO's 2008 classification of hematopoietic cancers (Swerdlow et al., 2008; Campo et al., 2011), in combination with all 453 known CD molecules, subtypes and isoforms were queried in PubMed as follows: “[cell] AND [CD molecule]” (Supplementary Figure 1). In addition to this search, all known aliases for both cells and CD molecules were queried. This resulted in a large number of hits, with only a minor fraction containing relevant experimental data. To facilitate efficient extraction of the relevant experimental data, we employed a text mining-based approach to classification of article abstracts as described in detail by Olsen et al. (2013). Initially, a small random set of abstracts was manually classified as either containing human CD molecule expression data or not. Of this set, we randomly selected 200 positive and 200 negative abstracts, which were then used to train a naive Bayes classifier. The classifier was subsequently applied to the remaining abstracts and those deemed positive for CD protein expression data were manually examined and relevant data extracted.

Most of the useful data we extracted were generated using flow cytometry of different varieties. In many instances, particularly in data published pre-1990, quantitative expression of CD molecules was not available. In these instances a binary “yes/no” designation was assigned to the cell/CD molecule data entry.

mRNA expression data analyzed here was generated by Novershtern et al. (2011) and downloaded through the Differentiation Map (DMAP) Portal (http://www.broadinstitute.org/dmap/). Novershtern and colleagues measured mRNA expression in 38 purified populations of human hematopoietic cells. Two of these cells (HSC CD133^+^ CD34_dim_ and HSC CD38^−^ CD34^+^) are normally considered to be identical cells in humans (Doulatov et al., 2012). Thus, in the following analyses we consider these two cells as one cell, namely the “hematopoietic stem cell.”

### CD molecule protein and mRNA expression data for half-live comparison

Analysis of CD protein mRNA and protein half-lives were based on data generated by Schwanhäusser et al. (2011), who quantified absolute levels of mRNA and protein in 5500 genes and 6445 proteins, as well as their degradation velocity in the mouse fibroblast cell-line NIH3T3. Using the “AnnotationDbi” package (Pages et al., 2008) for R version 3.1.0, all mouse CD proteins registered in the UniProt database were extracted from the dataset. From this dataset, we extracted the available 30 CD molecule genes for which both mRNA and protein expression data was measured. To test for robustness of the discovered values, a Monte Carlo analysis using 10,000 random samples of 30 random protein values extracted from the same dataset was applied.

### Clustering analyses

Clustering hematopoietic cells based on mRNA expression was performed using hierarchical clustering (Johnson, 1967) on the expression matrix of 37 cells and their expression of 35,459 mRNAs. Clustering based on CD molecule expression was likewise performed by hierarchical clustering on the same 37 cells and their expression of 261 CD molecules. Clustering and dendrograms were generated using the functions “hclust” and “dendrogram” in the R package “stats.” Hierarchical clustering and subsequent heat map representation was performed on the output from the PubMed searches performed in order to map data availability in the primary literature.

### Network representation

Network representation of cell similarity based on expression of CD molecules were generated by parsing all collected data into a Geography Markup Language (GML) format and imported into Cytoscape (Saito et al., 2012). Nodes represent hematopoietic cells and edges between nodes indicate protein level expression of a CD molecule in common between the two cells. The thicker the edges, the more CD molecules are expressed on both cells and thus provide a visual summary of the degree of commonality between the examined hematopoietic cells.

### Gene enrichment analysis of CD molecules

Two different background sets were used for the enrichment analysis: one for the general surface marker analysis where all the known human membrane proteins were used. These where extracted from UniProt (*n* = 2884). For analysis of various CD molecule subsets, we used all CD molecule genes including subtypes (*n* = 453) as the background set. Of these, only 397 currently have a gene name assigned. The hematopoietic cell types were divided into eight representative groups: B-cell lineage, T-cell lineage, NK cells, dendritic cells, macrophages, granulocytes, monocytes, and red blood cells & platelets (Supplementary Table 1), from which we derived the union of CD molecules expressed in the each subgroup and used these as gene sets in the enrichments analysis. Furthermore, we created a number of CD molecule subsets from the union of cell types found in the cluster analysis (Supplementary Table 2).

For evaluating the collective annotation of all CD genes as compared to the whole genome, GO::TermFinder, employing the GOA GO Slim ontology, was used (Boyle et al., 2004). This package contained 196 of the 397 CD genes mentioned above. All results are supplied in Supplementary Table 3.

The enrichment analysis was performed using GOrilla (Eden et al., 2009), which employs a standard hypergeometric test to estimate significantly enriched GO terms from a given subset of genes compared to a given background. The gene-ontology database in GOrilla is updated weekly (updated on Jul 12, 2014 for these analyses) ensuring an up to date gene ontology annotation of the input gene sets. We constrained our analyses only to include GO-terms for Biological Processes in Humans. All *p*-values were corrected for multiple testing to control the false discovery rate (FDR) as described by Benjamini and Hochberg (1995).

## Results and discussion

### Data collection

Almost 2 million PubMed searches were automatically performed as a result of the combined searches of all known hematopoietic cells and all known CD molecules, including aliases for both cells and CD molecules. Naive Bayes classification yielded reasonable performance for classification (accuracy = 0.78, sensitivity = 0.91, specificity = 0.65) and reduced the literature corpus to 1561 articles classified to contain the CD molecule expression data on human hematopoietic cells. Data from the articles available to us were manually extracted and resulted in 6153 data points containing the expression of 305 CD molecules on 206 hematopoietic cells from 1223 literature sources.

### Knowledge gaps in the immunophenotyping of blood cells

**Figure 1:**
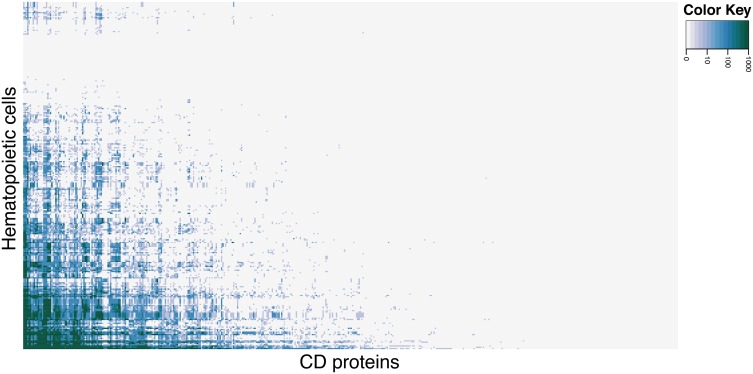
**Heat map of CD molecule expression data availability in primary literature**. PubMed was queried with combinations of each CD molecule with each hematopoietic cell (including common aliases for both). Light gray cells in the heat map correspond to 0 hits in PubMed for the cell and CD molecule combination, the shades of blue corresponds to 10–100 hits (light to dark), and green corresponds to 1000+ hits. See Supplementary Figure 1 for high-resolution figure with row and column names.

To identify hematopoietic cells and CD molecules for which data is currently lacking, we explored the primary literature via PubMed, registering the number of hits each cell type in combination with all CD molecules received. Not all searches yielded hits, which serves as an indicator of the state of experimental characterization of CD molecules on hematopoietic cells. Figure 1 shows a clustered heat map of PubMed hits for each cell (rows) and CD molecule (columns) search combination (see Supplementary Figure 1 for a high resolution version of the heat map with cells and CD molecules listed). The heat map (color scale from light gray for 0 hits to green for 1000+ hits) shows that only approximately 17% of all hematopoietic cell and CD molecule combinations have been reported in the primary literature. Only eight cell types had hits for 50 percent or more CD proteins.

Among the best characterized hematopoietic cells are T and B cell lineages, macrophages, plasma cells, stem- and progenitor cells as well as a number of cancers such as acute myeloid leukemia's (AML) and mature B-cell cancers. Under- or uncharacterized cells are mostly rare disease states including subtypes of AML and B lymphoblastic leukemia/lymphoma based on genetic rearrangements, which were only recently introduced in WHO's 2008 classification. Some eosinophil and basophil subtypes as well as CD4+ T follicular helper cell were surprisingly under-characterized. In total, 30 cell-types had no information available at all. Still, the number of articles published concerning the expression of a specific CD molecule on a specific cell is not necessarily reflective of our knowledge of the given cell. If no hits are found for a given cell/marker search, this could mean one of two things: (1) nobody has measure the expression of the marker on the cell yet, or (2) it could indicate that the marker is actually not expressed on the given cell, as negative results are rarely published. Tables 1, 2 highlights the best and least characterized cell types.

**Table 1:** **Ten best characterized cell types, as judged by available CD molecule data in the primary literature**.

**Name**	**Articles**	**Unique CD proteins**
T cell	334904	358
B cell	94993	312
Macrophage	99213	310
Plasma cell	47418	297
Plasmacytoid dendritic cell	65196	292
Hematopoietic stem cell	44287	253
Erythrocyte	17614	251
Neutrophil	38079	241
Neutrophil band cell	32312	236
Common myeloid progenitor	9708	232

*Numbers indicate found articles and surface markers*.

**Table 2:** **Ten least characterized cell types, as judged by available CD molecule data in the primary literature**.

**Name**	**Articles**	**Unique CD proteins**
Myeloid proliferations related to Down syndrome	2	3
Pro-NK cell	2	3
Myeloid and lymphoid neoplasms associated with PDGFRA rearrangement	9	3
Myelodysplastic syndrome, unclassified	3	4
Myelodysplastic/myeloproliferative neoplasm, unclassifiable	3	4
Disseminated juvenile xanthogranuloma	5	4
B lymphoblastic leukemia/lymphoma with recurrent genetic abnormalities	4	5
Myeloid and lymphoid neoplasms associated with FGFR1 abnormalities	13	5
Basophil-mast cell progenitor	5	6
Refractory cytopenia with unilineage dysplasia	6	6

*Numbers indicate found articles and surface markers*.

Of the 490 CD molecules queried in PubMed, 88 molecules yielded no hits at all when the search was combined with hematopoietic cell types. Upon inspection of these 88 markers, 8 were found to be provisional markers that would make little sense to screen for and 67 markers, while expressed in other tissues, have not been reported to be expressed on hematopoietic cells. A subset of 21 markers, although not reported to be expressed on hematopoietic cells in the primary literature, were listed as expressed in the secondary literature, such as proceedings, primarily from the Human Cell Differentiation Molecules (HCDM) workshops (http://www.hcdm.org/), but also from literature reviews and educational books.

### CD molecule mRNA and protein half-lives

If CD molecules are to be used for immunophenotyping or therapy, it is essential to know the expression and half-lives of these proteins, and it is of general interest to know the expression and half-life of the corresponding mRNAs. A number of studies have attempted to correlate mRNA levels to protein expression, hoping to model protein expression from high-throughput genomics methods, but poor correlation (Gry et al., 2009) and difficulties assessing membrane proteins (Sprenger and Jensen, 2010) hamper these attempts.

**Figure 2:**
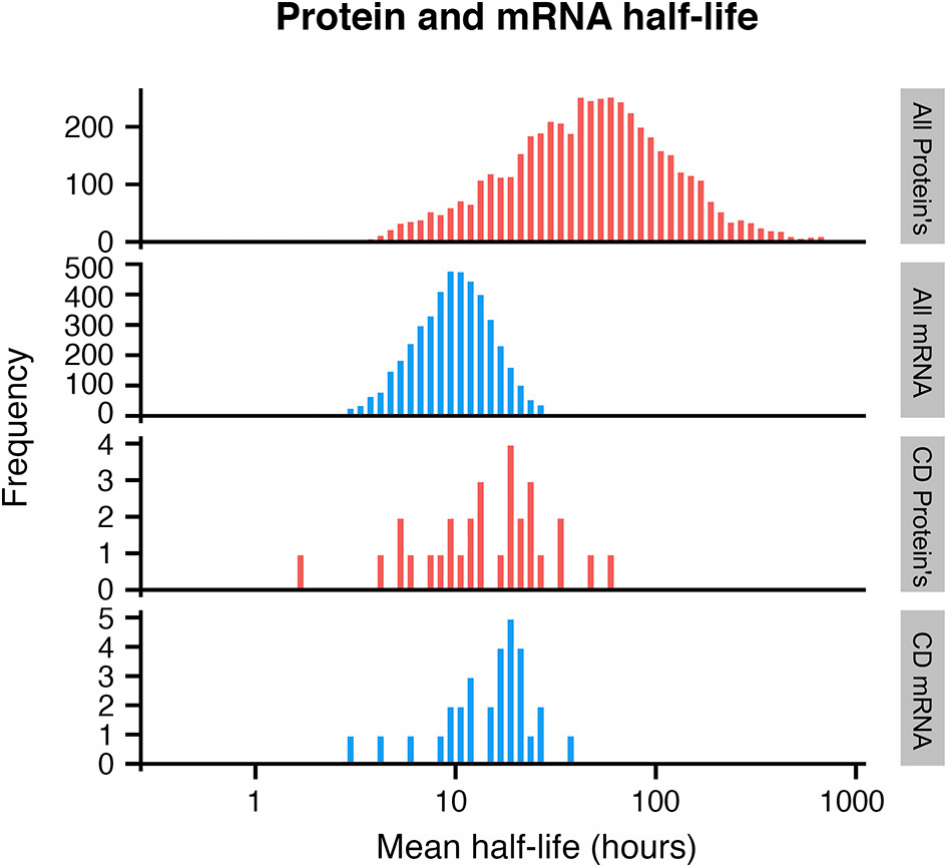
**Histogram of protein and mRNA half-lives in mammalian cells, based on Schwanhäusser et al. (2011)**. A subset of CD molecules for which data was available show faster turnover of membrane proteins (bottom 2 panels), when compared to all analyzed proteins (top 2 panels).

Exploring publicly available data, we therefore sought to evaluate the half-life of CD mRNA and protein. Unfortunately, very little quantitative expression data for CD molecules was available even in the largest dataset available with over 6000 mRNA and protein pairs measured (Schwanhäusser et al., 2011). Expression for 30 CD mRNA and protein pairs were found in total and these were compared with the global mRNA and protein half-lives. While the average global mRNA and protein half-life was 9 and 46 h, respectively, CD mRNA and proteins half-lives were on average 18 and 16 h for the 30 CD mRNAs and proteins, respectively (Figure 2). Although a small subset was used, a Monte Carlo simulation indicated that this subset could not have arisen randomly from the data used (Supplementary Figure 2).

This indicates that for CD molecules, there is a closer relationship between mRNA and protein levels over time, and that CD proteins have a much shorter half-life than the majority of cellular proteins. Cellular proteins are removed either by degradation, which is an active process, or by dilution which occurs when cells divide (Eden et al., 2011). Since dilution is a global event, affecting all proteins, one possible explanation for the shorter half-life of CD proteins could be that these proteins are under more active regulation in the cells.

### Defining the biological functions of CD molecules

We also examined up-regulated biological functions of the CDome as compared to the whole genome. We compared the enriched biological processes undertaken by genes within the CDome, and correlated this to the genome-wide use of these processes by identifying GO terms statistically over-represented in our subset compared to the genome. Not surprisingly, CD genes were significantly more involved in responses to stimuli (80 vs. 29%), regulation of cell behavior (73 vs. 40%), cell communication (62 vs. 22%) and cell differentiation (23 vs. 11%) (Supplementary Table 3).

**Figure 3:**
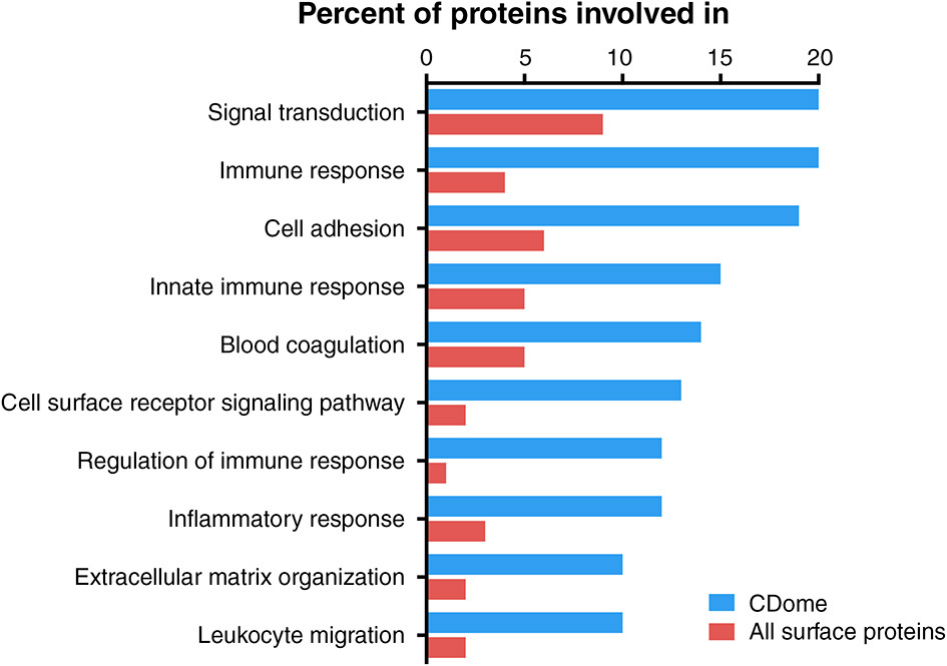
**Functional characterization of CD proteins, compared to all other UniProtKB annotated surface proteins based on GO terms**. Proteins within the CDome were found to be involved in signal transduction, immune response and adhesion processes to a higher degree than other surface proteins.

In order to examine if CD molecules differ from other surface proteins, we compared GO terms for CD proteins to GO terms for all 2884 known membrane proteins currently annotated in UniProt. We found 78 enriched GO-terms (*p*-value < 10^−10^, FDR < 10^−9^), most within the superfamilies of “immune system process,” “biological regulation” and “response to stimulus.” Most enriched processes were those involved in cell proliferation, leukocyte activation, response to cytokines and cellular insults, as well as regulation thereof. We then enumerated the total number of CD proteins involved in each of the biological processes, and compared those with all other membrane proteins (Figure 3). The proteins of the CDome were found to be involved in signal transduction, immune response and adhesion processes to a much higher degree than other surface proteins.

Taken together these results confirm that the CDome represents a broad class of biological functions, including those expected to be enriched in hematological cells, but also in a number of broader “housekeeping” cellular processes such as adhesion, cell signaling and proliferation. This implies that the CDome can be used broadly to assess the functional state of blood cells.

### Analysis of functions of CD molecule sets expressed on specific subsets

Data completeness remains a major obstacle for accurate analysis and, as described above, not all hematological cell-types are well characterized with regards to their expression of CD molecules. To test whether we could accurately describe cell types based solely on their CDome, we grouped 104 non-disease blood cells into the following categories: B-cells, T-cells, NK cells, dendritic cells, macrophages, granulocytes, monocytes and red blood cells (Supplementary Table 1). During our data collection process, we found data for 42 different CD molecules for each group on average. Using these data we performed a GSEA, comparing the CD molecules expressed on the cells of each category with all known membrane proteins as the background set. In Table 3, we have highlighted the functions found to be enriched in each group. For NK cells and macrophages, it was not possible to find enriched processes, but in the other groups a number of functions known to be specific for these cell types resulted. This suggests that the CD molecule expression data we have collected, serve as an indicator of hematopoietic cells function in general, and is of a quality that allows for further analyses.

**Table 3:** **Enriched GO term's based on the expression patterns of CD proteins can be used to differentiate cellular subtypes within the hematopoietic system**.

**Subset type**	**Unique CD proteins**	**GO-term summary**		
		**Description**	***P*-value**	**FDR**
B-cell lineage	69	Cellular process	2.67E-06	9.75E-03
		Regulation of inflammatory response	4.06E-06	7.42E-03
		Biological regulation	6.18E-05	7.54E-02
		Positive regulation of immune system process	9.68E-05	8.85E-02
T-cell lineage	49	Leukocyte migration	5.10E-07	1.87E-03
		Regulation of inflammatory response	1.25E-06	2.29E-03
		Cellular response to stimulus	1.54E-06	1.88E-03
		Locomotion	1.15E-05	4.67E-03
		Cell surface receptor signaling pathway	4.31E-06	3.15E-03
NK cells	20	None		
Dendritic cells	44	Response to molecule of bacterial origin	1.26E-06	4.61E-03
		Response to lipopolysaccharide	4.09E-06	7.48E-03
		Regulation of I-kappaB kinase/NF-kappaB signaling	7.95E-05	7.27E-02
Macrophages	35	None		
Granolocytes	29	Leukocyte migration	5.14E-06	1.88E-02
		Response to chemical	4.34E-05	7.94E-02
		Response to external stimulus	5.07E-05	6.17E-02
Monocytes	76	Cellular response to biotic stimulus	7.07E-06	2.58E-02
		Cellular response to molecule of bacterial origin	7.07E-06	1.29E-02
		Response to external stimulus	7.50E-06	9.14E-03
		Response to lipopolysaccharide	1.20E-05	8.77E-03
Red blood cells & platelets	14	Platelet activation	4.38E-08	1.60E-04
		Coagulation	7.44E-06	4.54E-03
		Hemostasis	7.44E-06	3.89E-03
		Cell adhesion	1.85E-04	6.14E-02
		Regulation of response to wounding	2.76E-04	7.76E-02

### Clustering hematopoietic cells by CD mRNA and protein expression

**Figure 4:**
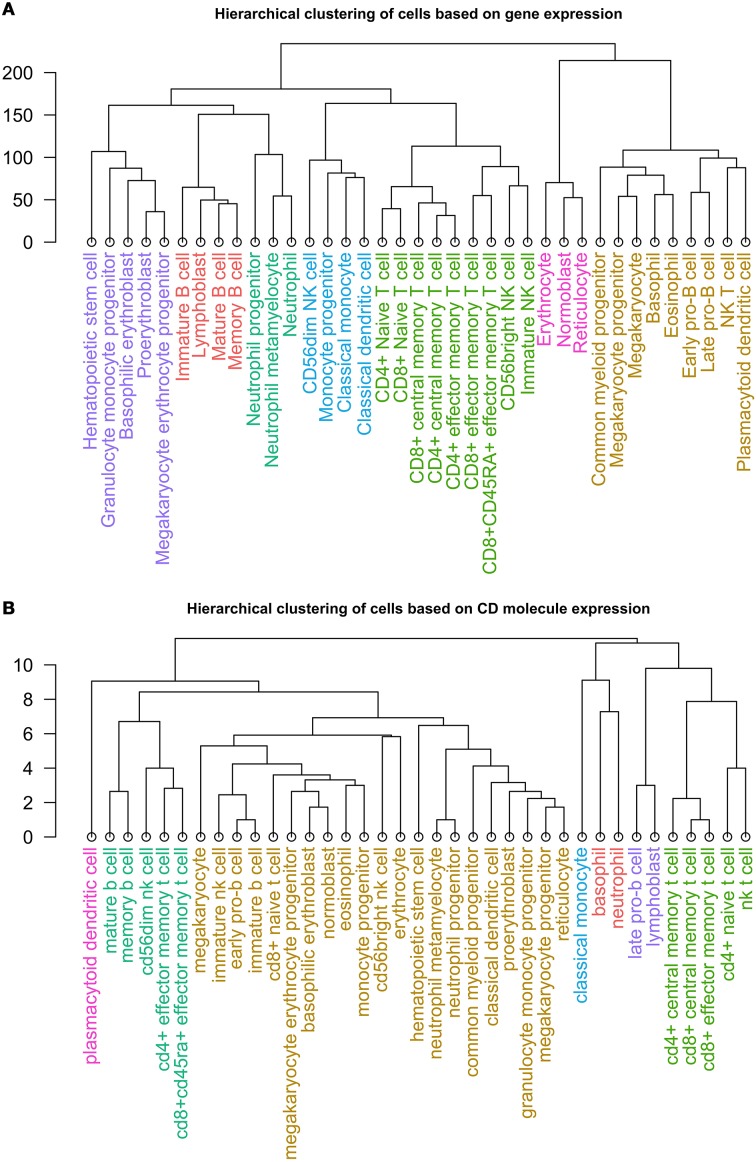
**Dendrogram of the clusters resulting from hierarchical clustering of 37 non-diseased hematopoietic cells by (A) expression of ~35,000 mRNAs and (B) expression of 305 CD protein**.

Lastly, we clustered healthy cell types according to expression of 305 CD molecules, and compared the results to those achieved by clustering by expression of approximately 35,000 mRNAs from individual hematopoietic cells (Novershtern et al., 2011). The hierarchical clustering of the cells by mRNA expression revealed seven distinct clusters (Figure 4A), which resembled groups one would expect following current concepts of hematopoiesis (Doulatov et al., 2012).

**Figure 5:**
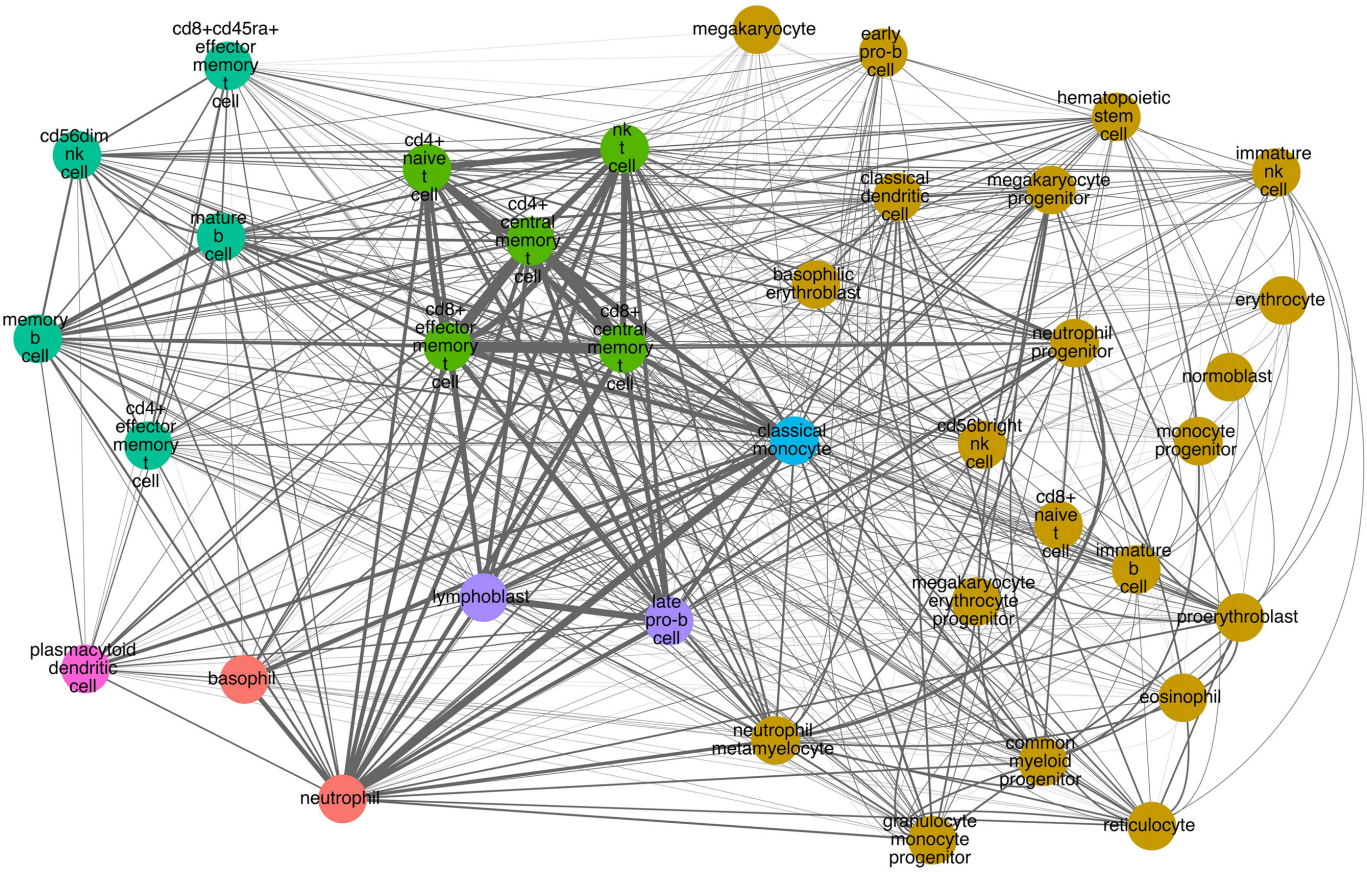
**CD molecule commonality graph on selected hematopoietic cells**. Nodes correspond to cells. Colors correspond to the hierarchical clustering based on CD molecule expression (Figure 4B). Edges signify one or more markers expressed on both cells. Edge thickness corresponds to the number of markers in common (the thicker the edge, the more markers in common between the two cells).

Similarly, we performed hierarchical clustering of the cells by CD molecule expression, which also revealed seven clusters (Figure 4B, Supplementary Table 2). These clusters, however, were quite different from the mRNA expression clusters. Using GSEA, we compared the individual clusters against all membrane proteins, and found a high overlap between the biological functions represented in each cluster (Supplementary Table 4). To explore this further, we constructed a cell-cell interaction network based on CD protein expression (Figure 5). Most cells appear to have at least one CD protein in common, while some of the more distinct clusters (for example, cluster 7 consisting of CD4+ naive T cells, CD4+ central memory T cells, CD8+ central memory T cell, CD8+ effector memory T cells, and NK T cells, highlighted in bright green on Figures 4B, 5) are heavily connected and have expression of a large number of CD proteins in common. While the protein cluster found could be a consequence of real biological differences at the protein level, at this point, it may also be attributed to incomplete CD expression data.

## Conclusions

Detailed knowledge about the membrane proteome is needed if we are to take full advantage of new therapies such as monoclonal antibodies and immunotherapy. There are two key issues with CD protein data availability, and consequently thorough characterization of the CDome: (1) technical obstacles to high throughput measurements of membrane proteins, and (2) the lack of an appropriate data resource for storing and accessing existing experimental data. To compensate for this, we collected 6153 CD protein expression data points from 1223 literature sources covering over 67% (305 of 453) of all CD molecules and more than 73% (206 of 279) of all hematopoietic cells. Data incompleteness may be attributed to the unstructured *ad hoc* data generation, the lack of standardized data storage, the fact that not all CD proteins are expressed on hematopoietic cells and that emerging and rare forms of cancer are still poorly described in the primary literature. To take full advantage of membrane proteins such as those within the CDome, increased effort should be invested in characterizing the surface proteome of cells—both in generating data in the wet lab by utilizing either mass cytometry or membrane mass spectometry, and cleaning, organizing, and storing data computationally.

Exploring mRNA and protein half-lives of a small set of CD molecules, we find indications that the half lives of CD mRNA and proteins correlate better than mRNA and proteins in general, due to a much shorter half-life of CD proteins. This indicates that CD protein expression is regulated more actively than intra-cellular proteins in general, underlining the need to characterize CD protein expression broadly across hematopoietic cells to facilitate efficient use of CD proteins for therapeutics.

We find that CD molecule functions are mainly associated with immunological processes, but also cover a range of cellular functions used by all cells. This is perhaps not surprising, as CD molecules have historically been used to primarily characterize cells within the immune system, but it may also indicate that CD protein expression may provide more specific functional classification of cells than expression of large set of differentially expressed genes (or all genes), which is often used in ontology studies.

To further investigate this notion, we clustered 37 hematopoietic cells by mRNA expression data, which yielded seven distinct clusters corresponding to what is commonly expected from hematopoiesis. Interestingly, when clustering the same cells by CD protein expression, we find that they cluster into seven quite different clusters. These clusters could perhaps give new insights functional groupings of cells, however more protein expression data is needed and a clustering on all hematopoietic cell types should be performed to further shape this hypothesis.

Overall, this article demonstrates that in order to drive advances within therapeutic hematology and aid basic research with cell stratification, a concerted effort must be made to better characterize the membrane proteome of cell types, and a central data resource for surface protein expression on cellular level should be established. Our analyses show that CD molecules are excellent therapy targets as they are well-characterized and control important biological function in blood cells, but also that a more systematic characterizing and organization of the cellular CDome should be retroactively established to accelerate further advances in the field.

### Conflict of interest statement

The authors declare that the research was conducted in the absence of any commercial or financial relationships that could be construed as a potential conflict of interest.
