# Supplementary material for: Characterizing the human hematopoietic CDome
Source: Front Genet. 2014 Sep 25;5:331. doi: 10.3389/fgene.2014.00331 (PMC4174859; doi:10.3389/fgene.2014.00331)
Supplement: Supplementary file 1 [file Table1.DOCX]

**Barnkob et al, Supplementary Table 1-4:**

| **Supplementary Table 1.** Subsets of cells. | |
| --- | --- |
| **Subset** | **Cells** |
|  |  |
| B-cell lineage | Pre-B cell, Early pro-B cell, Late pro-B cell, Large pre-B cell, Small pre-B cell, Immature B cell, B cell, Mature B cell, Lymphoblast, Memory B cell, Plasma cell |
| T-cell lineage | Pre-T cell, Pro-T cell, Pre-T cell, Thymocyte, Early double-negative T cell, Late double-negative T cell, Early double-positive T cell, Late double-positive T cell, T cell, Gamma delta T cell, CD4+ Naive T cell, CD8+ Naive T cell, NK T cell, CD4+ helper T cell, CD4+ Th1 T cell, CD4+ Th2 T cell, CD4+ Th17 T cell, CD4+ T follicular helper cell, CD4+ regulatory T cell, CD4+ effector memory T cell, CD4+ central memory T cell, CD8+ cytotoxic T cell, CD8+ effector memory T cell, CD8+CD45RA+ effector memory T cell, CD8+ central memory T cell, Tumor-infiltrating lymphocyte |
| NK Cells | Pro-NK cell, Pre-NK cell, Immature NK cell, CD56bright NK cell, CD56dim NK cell |
| Dendritic Cells | Common dendritic progenitor, Plasmacytoid dendritic cell, Classical dendritic cell |
| Macrophages | Macrophage, Tumor-associated macrophage, Tissue-resident macrophages, Adipose-tissue associated macrophage, Osteoclast, Bone-marrow macrophage, Microglia, Intestinal macrophage, Kupffer cell, Alveolar macrophage, Peritoneal macrophage, Pleural macrophage, Dermal macrophage, Marginal zone macrophage, Metallophilic macrophage, Red pulp macrophage, White pulp macrophage, Placental macrophage, Myeloid-derived suppressor cell |
| Granolocytes | Granulocyte monocyte progenitor, Basophil-mast cell progenitor, Mast cell progenitor, Mast cell, Basophil progenitor, Basophil myeloblast, Basophil promyelocyte, Basophil myelocyte, Basophil metamyelocyte, Basophil, Eosinophil progenitor, Eosinophil myeloblast, Eosinophil promyelocyte, Eosinophil myelocyte, Eosinophil metamyelocyte, Eosinophil, Neutrophil progenitor, Neutrophil myeloblast, Neutrophil promyelocyte, Neutrophil myelocyte, Neutrophil metamyelocyte, Neutrophil band cell, Neutrophil |
| Monocytes | Monocyte progenitor, Classical monocyte, Intermediate monocyte, Nonclassical monocyte |
| Red blood cells & platelets | Megakaryocyte erythrocyte progenitor, Megakaryocyte progenitor, Promegakaryocyte, Megakaryoblast, Megakaryocyte, Thrombocyte, Erythrocyte progenitor, Proerythroblast, Basophilic erythroblast, Polychromotophilic erythroblast, Normoblast, Reticulocyte, Erythrocyte |

|  |  |
| --- | --- |
| **Supplementary Table 2.** Cell clustering based on CD protein expression. | |
| **Cluster Number** | **Cells** |
|  |  |
| Cluster 1 | Plasmacytoid dendritic cell |
| Cluster 2 | Mature B cell, Memory B cell, CD56dim NK cell, CD4+ effector memory T cell, CD8+CD45RA+ effector memory T cell |
| Cluster 3 | Megakaryocyte, Immature NK cell, Early pro-B cell, Immature B cell, CD8+ Naive T cell, Megakaryocyte erythrocyte progenitor, Basophilic erythroblast, Normoblast, Eosinophil, Monocyte progenitor, CD56bright NK cell, Erythrocyte, Hematopoietic stem cell, Neutrophil metamyelocyte, Neutrophil progenitor, Common myeloid progenitor, Classical dendritic cell, Proerythroblast, Granulocyte monocyte progenitor, Megakaryocyte progenitor, Reticulocyte |
| Cluster 4 | Classical monocyte |
| Cluster 5 | Basophil, Neutrophil |
| Cluster 6 | Late pro-B cell, Lymphoblast |
| Cluster 7 | CD4+ central memory T cell, CD8+ central memory T cell, CD8+ effector memory T cell, CD4+ Naive T cell, NK T cell |

| **Supplementary Table 3.** Analysis of GO terms associated with CD genes | | | |
| --- | --- | --- | --- |
| **GO Term Name (ID)** | **Genes** | **Percentage of CD Genes** | **Percentages in all Genes** |
|  |  |  |  |
| Response to stimulus ( GO:0050896 ) | ABCG2, ART1, BMPR1A, BST2, BTLA, BTN3A1, C5AR1, CCR1, CCR5, CCR9, CD101, CD109, CD160, CD164, CD177, CD180, CD19, CD1A, CD1D, CD200, CD207, CD24, CD244, CD247, CD27, CD274, CD28, CD300A, CD300C, CD300E, CD300LB, CD33, CD40, CD46, CD47, CD55, CD58, CD72, CD79A, CD79B, CD80, CD83, CD84, CD8B, CD96, CEACAM1, CEACAM6, CEACAM8, CR1, CR2, CRTAM, CTLA4, CXCR1, CXCR3, CXCR4, CXCR5, CXCR6, ENTPD1, EPCAM, F3, FASLG, FCAR, FCGR1A, FCGR2A, FCGR2B, FCGR2C, FCGR3A, FCGR3B, FZD10, FZD4, FZD9, GP1BA, GP1BB, GP5, GP9, ICAM4, ICOS, IFITM1, IGLL1, IL10RB, IL13RA2, IL15RA, IL17RA, IL18R1, IL21R, IL2RA, IL6ST, INSR, ITGA1, ITGAD, ITGAE, ITGAM, ITGAV, JAM2, KDR, KIR2DL1, KIR2DL2, KIR2DL4, KIR2DS1, KIR2DS2, KIR2DS4, KIR2DS5, KIR3DL1, KIR3DL2, KIT, KLRB1, LAG3, LILRA1, LILRA2, LILRA3, LILRA5, LILRB1, LILRB2, LILRB3, LILRB4, LILRB5, LY75, MCAM, NCR1, NCR2, NCR3, PDCD1LG2, PECAM1, PROCR, PTGDR2, PTPRJ, PVR, PVRL1, PVRL2, S1PR1, SELL, SELPLG, SIGLEC1, SIGLEC9, SIRPA, SIRPG, SLAMF8, TEK, THBD, TLR2, TLR4, TLR8, TLR9, TNFRSF10A, TNFRSF10D, TNFRSF11A, TNFRSF13C, TNFRSF17, TNFRSF1B, TNFRSF21, TNFRSF4, TNFSF10, TNFSF11, TNFSF13B, TNFSF14, TNFSF8, TREM1. | 80.10% | 29.31% |
| Cellular process ( GO:0009987 ) | ABCG2, ART1, BMPR1A, BST2, BTLA, BTN3A1, C5AR1, CCR1, CCR5, CCR9, CD101, CD109, CD160, CD164, CD177, CD180, CD19, CD1D, CD24, CD244, CD247, CD248, CD27, CD274, CD28, CD300A, CD300C, CD320, CD33, CD40, CD46, CD47, CD52, CD55, CD58, CD72, CD79A, CD79B, CD80, CD83, CD84, CD8B, CD93, CEACAM1, CEACAM5, CEACAM6, CR1, CR2, CRTAM, CTLA4, CXCR1, CXCR3, CXCR4, CXCR5, CXCR6, ENTPD1, EPCAM, F3, FASLG, FCGR1A, FCGR2A, FCGR2B, FCGR2C, FCGR3A, FUT4, FZD10, FZD4, FZD9, GP1BA, GP1BB, GP5, GP9, ICAM4, ICOS, IFITM1, IGSF8, IL10RB, IL13RA2, IL15RA, IL17RA, IL18R1, IL21R, IL2RA, IL6ST, INSR, ITGA1, ITGAD, ITGAE, ITGAM, ITGAV, JAM2, KDR, KIR2DL1, KIR2DL4, KIR2DS1, KIR2DS2, KIR2DS5, KIR3DL1, KIT, KLRB1, LAG3, LILRA1, LILRA2, LILRA3, LILRB1, LILRB2, LILRB3, LILRB4, LILRB5, NCR1, NCR2, NCR3, PDCD1LG2, PECAM1, PTGDR2, PTGFRN, PTPRJ, PVR, PVRL1, PVRL2, PVRL3, RHAG, S1PR1, SELL, SELPLG, SIGLEC1, SIGLEC6, SIGLEC9, SIRPA, SIRPG, SLAMF8, SLC4A1, TEK, THBD, TIGIT, TLR2, TLR4, TLR8, TLR9, TNFRSF10A, TNFRSF10D, TNFRSF11A, TNFRSF13C, TNFRSF17, TNFRSF1B, TNFRSF21, TNFRSF4, TNFRSF9, TNFSF10, TNFSF11, TNFSF13B, TNFSF14, TNFSF8, TREM1 | 78.57% | 65.69% |
| Regulation of biological process ( GO:0050789 ) | BMPR1A, BST2, BTLA, BTN3A1, C5AR1, CCR1, CCR5, CCR9, CD101, CD109, CD160, CD164, CD180, CD19, CD1D, CD200, CD24, CD244, CD247, CD248, CD27, CD274, CD28, CD300A, CD300C, CD320, CD33, CD40, CD46, CD47, CD55, CD58, CD72, CD79A, CD79B, CD80, CD83, CD8B, CD96, CEACAM1, CEACAM5, CEACAM6, CR1, CR2, CRTAM, CTLA4, CXCR1, CXCR3, CXCR4, CXCR5, CXCR6, ENTPD1, EPCAM, F3, FASLG, FCGR1A, FCGR2A, FCGR2B, FCGR2C, FCGR3A, FZD10, FZD4, FZD9, GP1BA, GP1BB, GP5, ICAM4, ICOS, IFITM1, IL10RB, IL13RA2, IL15RA, IL17RA, IL18R1, IL21R, IL2RA, IL6ST, INSR, ITGA1, ITGAD, ITGAE, ITGAM, ITGAV, JAM2, KDR, KIR2DL1, KIR2DL2, KIR2DL4, KIR2DS1, KIR2DS2, KIR2DS5, KIR3DL1, KIR3DL2, KIT, KLRB1, LAG3, LILRA1, LILRA2, LILRA3, LILRB1, LILRB2, LILRB3, LILRB4, LILRB5, NCR1, NCR2, NCR3, PDCD1LG2, PECAM1, PROCR, PTGDR2, PTPRJ, PVR, PVRL1, PVRL2, S1PR1, SELL, SIGLEC1, SIGLEC9, SIRPG, SLAMF8, TEK, THBD, TIGIT, TLR2, TLR4, TLR8, TLR9, TNFRSF10A, TNFRSF10D, TNFRSF11A, TNFRSF13C, TNFRSF17, TNFRSF1B, TNFRSF21, TNFRSF4, TNFRSF9, TNFSF10, TNFSF11, TNFSF13B, TNFSF14, TNFSF8, TREM1 | 72.96% | 39.57% |
| Cell communication ( GO:0007154 ) | BMPR1A, BST2, BTLA, BTN3A1, C5AR1, CCR1, CCR5, CCR9, CD101, CD109, CD160, CD164, CD180, CD19, CD24, CD244, CD247, CD27, CD274, CD28, CD300A, CD300C, CD33, CD40, CD46, CD47, CD55, CD72, CD79A, CD79B, CD80, CD83, CD8B, CEACAM1, CEACAM6, CR1, CR2, CTLA4, CXCR1, CXCR3, CXCR4, CXCR5, CXCR6, ENTPD1, EPCAM, F3, FASLG, FCGR1A, FCGR2A, FCGR2B, FCGR2C, FCGR3A, FZD10, FZD4, FZD9, GP1BA, GP1BB, IFITM1, IL10RB, IL13RA2, IL15RA, IL17RA, IL18R1, IL21R, IL2RA, IL6ST, INSR, ITGA1, ITGAD, ITGAE, ITGAM, ITGAV, KDR, KIR2DL1, KIR2DL4, KIR2DS1, KIR2DS2, KIR2DS5, KIR3DL1, KIT, KLRB1, LAG3, LILRA1, LILRA2, LILRA3, LILRB1, LILRB2, LILRB3, LILRB4, LILRB5, NCR1, NCR2, PECAM1, PTGDR2, PTPRJ, PVRL1, PVRL2, S1PR1, SIGLEC1, SIGLEC6, SIGLEC9, SIRPG, TEK, THBD, TLR2, TLR4, TLR8, TLR9, TNFRSF10A, TNFRSF10D, TNFRSF11A, TNFRSF17, TNFRSF1B, TNFRSF21, TNFRSF4, TNFSF10, TNFSF11, TNFSF13B, TNFSF14, TNFSF8, TREM1 | 61.73% | 22.13% |
| Multicellular organismal process ( GO:0032501 ) | BMPR1A, BST2, BTN3A1, C5AR1, CCR1, CD164, CD177, CD1D, CD24, CD244, CD248, CD27, CD274, CD28, CD40, CD46, CD47, CD55, CD58, CD72, CD79A, CD80, CD83, CD84, CEACAM1, CR2, CRTAM, CTLA4, CXCR3, CXCR4, CXCR5, ENTPD1, EPCAM, F3, FASLG, FZD10, FZD4, FZD9, GP1BA, GP1BB, GP5, GP9, ICOS, IFITM1, IGSF8, IL15RA, IL17RA, IL18R1, IL2RA, IL6ST, INSR, ITGA1, ITGAM, ITGAV, JAM2, KDR, KIT, LAG3, LILRB1, LILRB2, LILRB3, LILRB4, MCAM, NCR3, PECAM1, PROCR, PSG1, PTPRJ, PVRL1, PVRL2, PVRL3, S1PR1, SELL, SELPLG, SIRPA, SIRPG, TEK, THBD, TIGIT, TLR2, TLR4, TLR8, TLR9, TNFRSF11A, TNFRSF13C, TNFRSF17, TNFRSF21, TNFRSF4, TNFRSF9, TNFSF10, TNFSF11, TNFSF8, TREM1 | 47.45% | 21.37% |
| Metabolic process ( GO:0008152 ) | ABCG2, ART1, BMPR1A, BST2, C5AR1, CCR1, CCR5, CD101, CD109, CD24, CD27, CD28, CD300A, CD320, CD40, CD46, CD52, CD55, CD80, CR1, CR2, CXCR1, CXCR3, CXCR4, ENTPD1, EPCAM, F3, FASLG, FUT4, FZD10, FZD4, GP1BA, GP1BB, GP5, GP9, IL6ST, INSR, ITGA1, ITGAV, KDR, KIT, LAG3, LILRB1, PTPRJ, RHAG, S1PR1, SLAMF8, SLC4A1, TEK, TLR2, TLR4, TLR8, TLR9, TNFRSF10A, TNFRSF11A, TNFRSF13C, TNFRSF1B, TNFRSF21, TNFRSF4, TNFSF10, TNFSF11, TNFSF14, TNFSF8 | 32.14% | 57.15% |
| Multicellular organismal development ( GO:0007275 ) | BMPR1A, BST2, C5AR1, CCR1, CD164, CD1D, CD24, CD248, CD27, CD28, CD40, CD46, CD72, CD79A, CD80, CD83, CEACAM1, CR2, CTLA4, CXCR3, CXCR4, CXCR5, EPCAM, F3, FASLG, FZD10, FZD4, FZD9, ICOS, IGSF8, IL15RA, IL18R1, IL2RA, IL6ST, INSR, ITGA1, ITGAM, ITGAV, KDR, KIT, LILRB1, LILRB2, LILRB3, LILRB4, MCAM, PECAM1, PTPRJ, PVRL1, PVRL3, S1PR1, TEK, THBD, TLR2, TLR4, TNFRSF11A, TNFRSF17, TNFRSF21, TNFSF10, TNFSF11, TNFSF8 | 30.61% | 15.43% |
| Macromolecule metabolic process ( GO:0043170 ) | ART1, BMPR1A, BST2, C5AR1, CCR1, CD109, CD24, CD27, CD28, CD300A, CD40, CD46, CD55, CD80, CR1, CR2, CXCR1, CXCR3, CXCR4, EPCAM, F3, FASLG, FUT4, FZD10, FZD4, GP1BA, GP1BB, GP5, GP9, IL6ST, INSR, ITGA1, ITGAV, KDR, KIT, LAG3, LILRB1, PTPRJ, S1PR1, TEK, TLR2, TLR4, TLR8, TLR9, TNFRSF10A, TNFRSF11A, TNFRSF13C, TNFRSF1B, TNFRSF4, TNFSF10, TNFSF11, TNFSF14, TNFSF8 | 27.04% | 37.67% |
| Transport ( GO:0006810 ) | ABCG2, BMPR1A, BST2, BTN3A1, CCR1, CCR5, CD24, CD247, CD27, CD274, CD300A, CD302, CD40, CD47, CD58, CD93, CRTAM, CXCR1, CXCR3, FCGR1A, FCGR2A, FCGR3A, FZD4, IL18R1, INSR, ITGAV, KIT, LILRB1, LILRB2, LY75, MRC1, PECAM1, PVRL1, RHAG, SIGLEC1, SLC4A1, TLR2, TLR4, TLR8, TLR9, TNFRSF11A, TNFRSF21, TNFRSF4, TNFRSF9, TNFSF11, TNFSF13B, TNFSF14 | 23.98% | 17.19% |
| Multi-organism process ( GO:0051704 ) | BST2, C5AR1, CCR5, CD160, CD180, CD1D, CD207, CD24, CD247, CD28, CD40, CD46, CD47, CD55, CD80, CD8B, CD93, CXCR4, CXCR6, FASLG, FCGR2B, IFITM1, IGSF8, IL2RA, IL6ST, INSR, ITGAV, KDR, KIT, LILRB1, LILRB2, PSG1, PVR, PVRL1, PVRL2, PVRL3, SLAMF8, THBD, TLR2, TLR4, TLR8, TLR9, TNFRSF11A, TNFRSF1B, TNFSF8 | 22.96% | 6.38% |
| Cell differentiation ( GO:0030154 ) | BMPR1A, CCR1, CD1D, CD24, CD27, CD28, CD46, CD72, CD79A, CD80, CD83, CEACAM5, CR2, CTLA4, CXCR4, EPCAM, FASLG, FZD10, FZD4, FZD9, IFITM1, IL15RA, IL18R1, IL2RA, IL6ST, ITGA1, ITGAM, ITGAV, KDR, KIT, LILRB1, LILRB2, LILRB3, LILRB4, PTPRJ, PVRL1, PVRL2, S1PR1, TEK, TLR2, TLR4, TNFRSF11A, TNFRSF21, TNFSF11, TNFSF8 | 22.96% | 10.78% |
| Cellular component movement ( GO:0006928 ) | BST2, C5AR1, CCR1, CCR5, CD177, CD24, CD244, CD248, CD300A, CD47, CD58, CD72, CD84, CEACAM1, CXCR1, CXCR3, CXCR4, CXCR5, F3, IFITM1, IGSF8, INSR, ITGA1, ITGAM, ITGAV, JAM2, KDR, KIT, PECAM1, PTPRJ, PVR, PVRL1, S1PR1, SELL, SELPLG, SIRPA, SIRPG, TEK, THBD, TLR2, TNFRSF11A, TNFSF11, TNFSF14, TREM1 | 22.45% | 5.38% |
| Cell death ( GO:0008219 ) | C5AR1, CD24, CD248, CD27, CD28, CD40, CEACAM5, CTLA4, CXCR3, CXCR4, F3, FASLG, IL2RA, IL6ST, ITGA1, ITGAV, KDR, KIT, LILRB1, SIGLEC1, TEK, TLR2, TNFRSF10A, TNFRSF10D, TNFRSF1B, TNFRSF21, TNFRSF4, TNFRSF9, TNFSF10, TNFSF14, TNFSF8 | 15.82% | 6.09% |
| Biosynthetic process ( GO:0009058 ) | ART1, BMPR1A, C5AR1, CD28, CD40, CD80, CXCR3, EPCAM, FASLG, FUT4, FZD4, INSR, ITGAV, KDR, KIT, LAG3, LILRB1, S1PR1, TLR2, TLR4, TLR8, TLR9, TNFRSF11A, TNFRSF13C, TNFRSF4, TNFSF11, TNFSF8 | 13.78% | 23.35% |
| Nucleobase, nucleoside, nucleotide and nucleic acid metabolic process ( GO:0006139 ) | ABCG2, BMPR1A, C5AR1, CD28, CD40, CD80, CXCR3, ENTPD1, EPCAM, FASLG, FZD10, FZD4, INSR, KIT, LILRB1, S1PR1, TLR2, TLR4, TLR9, TNFRSF11A, TNFRSF4, TNFSF11, TNFSF8 | 11.73% | 26.15% |
| Secretion ( GO:0046903 ) | BTN3A1, CCR1, CD274, CD300A, CD40, CD58, CRTAM, FZD4, KIT, LILRB1, PECAM1, TLR2, TLR4, TLR8, TLR9, TNFRSF11A, TNFRSF21, TNFRSF4, TNFRSF9, TNFSF11, TNFSF13B | 10.71% | 3.03% |
| Behavior ( GO:0007610 ) | CCR1, CXCR3, CXCR4, F3, FZD4, FZD9, KDR, KIT, PTPRJ, S1PR1, TNFSF14 | 5.61% | 2.01% |
| Extracellular structure organization ( GO:0043062 ) | CD47, ICAM4, ITGA1, ITGAD, ITGAE, ITGAM, ITGAV, JAM2, KDR, PECAM1 | 5.10% | 1.15% |
| Catabolic process ( GO:0009056 ) | ABCG2, CD40, ENTPD1, FUT4, FZD10, INSR, S1PR1, TNFRSF1B | 4.08% | 10.54% |

| **Supplementary Table 4**. Enriched GO Terms Across Cell Clusters. | | | | |
| --- | --- | --- | --- | --- |
| **Subset Type** | **Unique CD Proteins** | **GO-term summary** | | |
|  |  | *Description* | *P-value* | *FDR q-value* |
| Cluster 1 | 49 | immune system process | 9,39E-20 | 6,96E-16 |
|  |  | regulation of immune system process | 9,59E-12 | 3,56E-08 |
|  |  | positive regulation of immune system process | 3,32E-11 | 8,20E-08 |
|  |  | immune response | 9,71E-11 | 1,80E-07 |
|  |  | regulation of cytokine production | 3,11E-10 | 4,60E-07 |
|  |  | regulation of immune response | 3,19E-09 | 3,95E-06 |
|  |  | regulation of cytokine secretion | 1,11E-08 | 1,18E-05 |
|  |  | regulation of lymphocyte proliferation | 1,41E-08 | 1,30E-05 |
|  |  | regulation of protein secretion | 1,41E-08 | 1,16E-05 |
|  |  | lymphocyte activation | 1,64E-08 | 1,22E-05 |
| Cluster 2 | 59 | immune system process | 2,51E-24 | 1,86E-20 |
|  |  | positive regulation of immune system process | 3,80E-14 | 1,41E-10 |
|  |  | leukocyte migration | 1,51E-13 | 3,73E-10 |
|  |  | regulation of immune system process | 7,53E-13 | 1,39E-09 |
|  |  | defense response | 1,48E-12 | 2,19E-09 |
|  |  | immune response | 3,63E-12 | 4,48E-09 |
|  |  | cytokine-mediated signaling pathway | 5,23E-12 | 5,53E-09 |
|  |  | regulation of cell activation | 6,80E-12 | 6,29E-09 |
|  |  | regulation of lymphocyte activation | 1,38E-11 | 1,13E-08 |
|  |  | positive regulation of cell activation | 2,12E-11 | 1,57E-08 |
| Cluster 3 | 111 | immune system process | 5,93E-24 | 4,43E-20 |
|  |  | regulation of immune system process | 6,37E-16 | 2,38E-12 |
|  |  | leukocyte migration | 1,14E-14 | 2,83E-11 |
|  |  | response to cytokine | 8,82E-14 | 1,65E-10 |
|  |  | cellular response to cytokine stimulus | 1,41E-13 | 2,11E-10 |
|  |  | positive regulation of immune system process | 8,50E-13 | 1,06E-09 |
|  |  | extracellular matrix organization | 2,03E-12 | 2,16E-09 |
|  |  | extracellular structure organization | 2,03E-12 | 1,89E-09 |
|  |  | cytokine-mediated signaling pathway | 2,05E-12 | 1,70E-09 |
|  |  | coagulation | 2,42E-12 | 1,81E-09 |
| Cluster 4 | 104 | immune system process | 1,14E-32 | 8,51E-29 |
|  |  | response to external stimulus | 1,03E-19 | 3,85E-16 |
|  |  | response to external biotic stimulus | 6,12E-19 | 1,52E-15 |
|  |  | leukocyte migration | 1,59E-18 | 2,96E-15 |
|  |  | response to biotic stimulus | 2,60E-18 | 3,87E-15 |
|  |  | regulation of immune system process | 1,70E-17 | 2,11E-14 |
|  |  | positive regulation of immune system process | 1,97E-17 | 2,10E-14 |
|  |  | defense response | 7,01E-16 | 6,54E-13 |
|  |  | regulation of immune response | 1,58E-14 | 1,31E-11 |
|  |  | response to cytokine | 1,39E-13 | 1,04E-10 |
| Cluster 5 | 89 | immune system process | 4,42E-26 | 3,29E-22 |
|  |  | leukocyte migration | 2,52E-16 | 9,37E-13 |
|  |  | response to external biotic stimulus | 8,26E-15 | 2,05E-11 |
|  |  | response to biotic stimulus | 2,59E-14 | 4,82E-11 |
|  |  | response to cytokine | 1,28E-13 | 1,91E-10 |
|  |  | positive regulation of immune system process | 2,19E-13 | 2,72E-10 |
|  |  | response to external stimulus | 2,43E-13 | 2,58E-10 |
|  |  | cellular response to cytokine stimulus | 3,94E-13 | 3,66E-10 |
|  |  | regulation of immune system process | 4,47E-13 | 3,69E-10 |
|  |  | defense response | 7,38E-13 | 5,49E-10 |
| Cluster 6 | 72 | immune system process | 6,63E-19 | 4,91E-15 |
|  |  | regulation of immune system process | 2,43E-15 | 9,02E-12 |
|  |  | leukocyte migration | 4,44E-15 | 1,10E-11 |
|  |  | regulation of immune response | 2,67E-13 | 4,95E-10 |
|  |  | cellular component movement | 1,29E-12 | 1,91E-09 |
|  |  | positive regulation of immune system process | 4,38E-12 | 5,41E-09 |
|  |  | extracellular matrix organization | 2,81E-11 | 2,98E-08 |
|  |  | extracellular structure organization | 2,81E-11 | 2,60E-08 |
|  |  | locomotion | 3,05E-11 | 2,51E-08 |
|  |  | cellular response to cytokine stimulus | 3,44E-11 | 2,55E-08 |
| Cluster 7 | 121 | immune system process | 5,22E-39 | 3,88E-35 |
|  |  | regulation of immune system process | 2,95E-30 | 1,10E-26 |
|  |  | positive regulation of immune system process | 1,40E-28 | 3,46E-25 |
|  |  | regulation of lymphocyte activation | 8,10E-23 | 1,51E-19 |
|  |  | positive regulation of cell activation | 2,63E-22 | 3,92E-19 |
|  |  | leukocyte migration | 3,75E-22 | 4,66E-19 |
|  |  | cytokine-mediated signaling pathway | 1,49E-21 | 1,58E-18 |
|  |  | positive regulation of leukocyte activation | 1,88E-21 | 1,75E-18 |
|  |  | regulation of leukocyte activation | 2,91E-21 | 2,41E-18 |
|  |  | regulation of cell activation | 3,03E-21 | 2,25E-18 |
