# Supplementary material for: Characterizing the human hematopoietic CDome
Source: Front Genet. 2014 Sep 25;5:331. doi: 10.3389/fgene.2014.00331 (PMC4174859; doi:10.3389/fgene.2014.00331)
Supplement: Supplementary file 2 [file Image1.PDF]

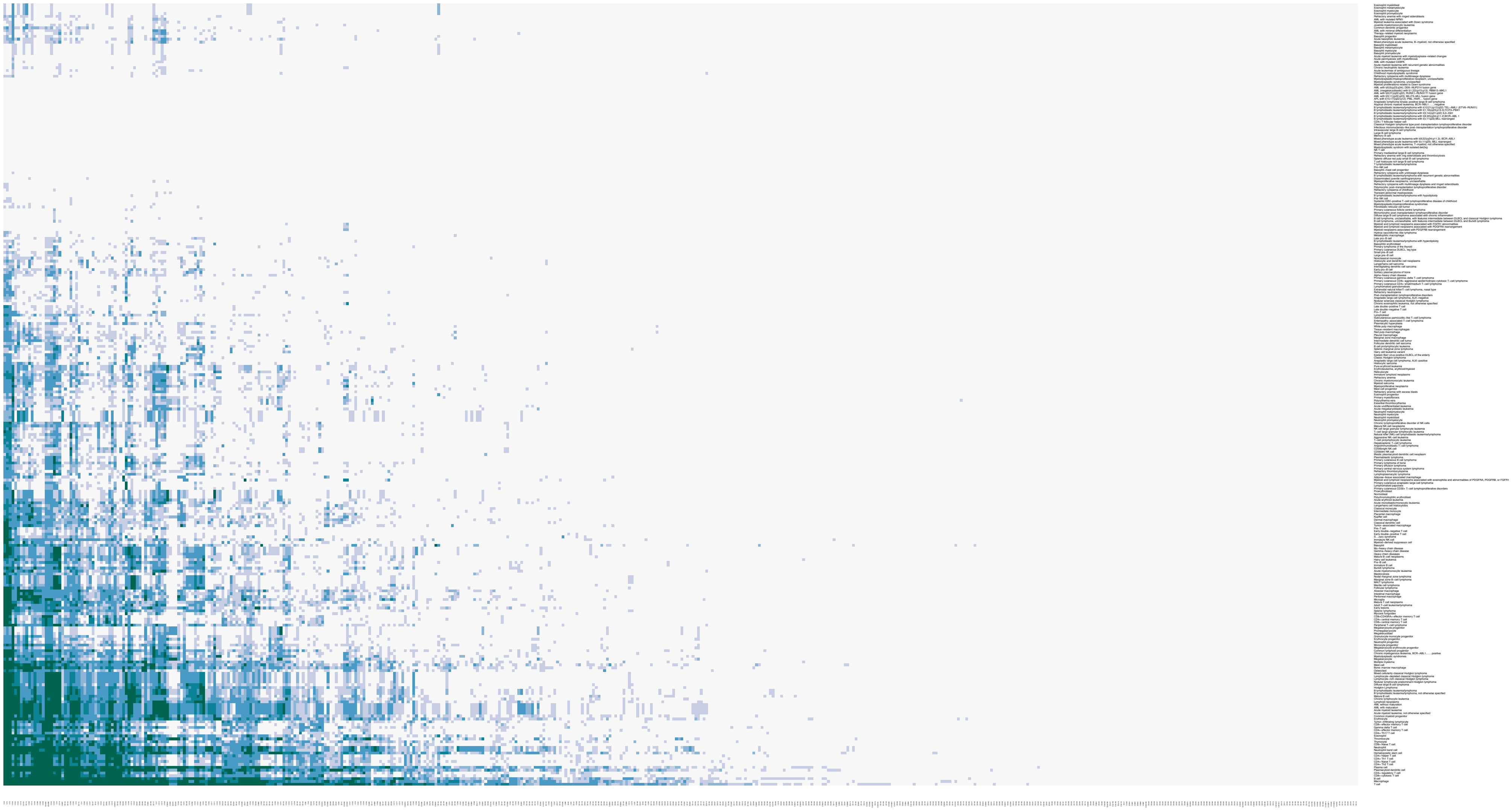

FIGURE 1 | Heat map of CD molecule expression data availability in primary literature. PubMed was queried with combinations of each CD molecule with each hematopoietic cell (including common aliases for both). Light gray cells in the heat map correspond to 0 hits in PubMed for the cell and CD molecule combination, the shades of blue corresponds to 10–100 hits (light to dark), and green corresponds to 1000+ hits. See Supplementary Figure 1 for high-resolution figure with row and column names.
