# Supplementary material for: Characterizing the human hematopoietic CDome
Source: Front Genet. 2014 Sep 25;5:331. doi: 10.3389/fgene.2014.00331 (PMC4174859; doi:10.3389/fgene.2014.00331)
Supplement: Supplementary file 3 [file Image2.PDF]

Supplementary Figure 2

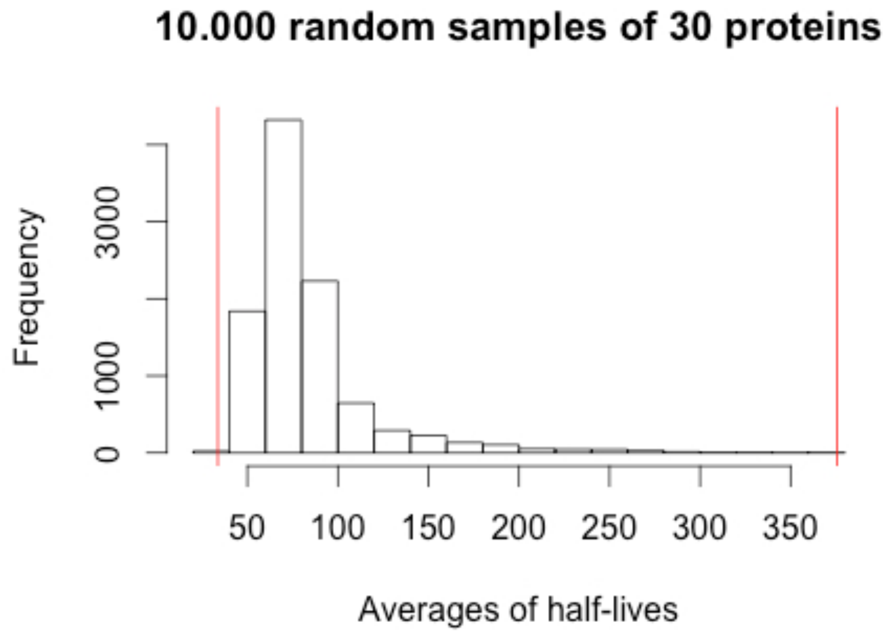

Supplementary Figure 2: Monte Carlo simulation to test for robustness of the calculated values within the small dataset of CD mRNA and protein expression (n = 30). 10,000 random samples of 30 proteins were extracted from the Schwanhäusser et al. 2011 dataset, and the average half lives are plotted. Left and right red line indicate minimum (33.68h) and maximum (375.75h) of found averages. None are equal to or lower than the average half-life of found set (17.81h).
